# Stroke and Thrombotic Events Associated With Concomitant Use of Oral Anticoagulants and Antiepileptic Drugs in the United States and Japan

**DOI:** 10.7759/cureus.83268

**Published:** 2025-04-30

**Authors:** Tomiko Sunaga, Ryo Yonezawa

**Affiliations:** 1 Clinical Epidemiology, Division of Applied Pharmaceutical Education and Research, Hoshi University, Tokyo, JPN; 2 Hospital Pharmaceutics, School of Pharmacy, Showa Medical University, Tokyo, JPN

**Keywords:** antiepileptic drug, drug interaction, oral anticoagulant therapy (oat), stroke, thrombotic event

## Abstract

Introduction: Direct oral anticoagulants (DOACs) are substrates of efflux P-glycoprotein (P-gp) transporters, and the hepatic enzyme cytochrome P450 plays an important role. Antiepileptic drugs (AEDs) may reduce absorption or increase the metabolism of DOACs, thereby reducing their antithrombotic efficacy. However, there is no evidence that concurrent use of metabolic inducers in patients taking DOACs is associated with an increased risk of stroke or thrombotic events.

Methods: We analyzed adverse event cases submitted to the Food and Drug Administration Adverse Event Reporting System (FAERS) from January 2010 to June 2023 and the Japanese Adverse Drug Event Report (JADER) database to April 2023. We compared the proportion of cases reporting thromboembolic and ischemic adverse events with the concomitant use of DOACs and first-generation drugs, such as phenytoin, metabolically inducing AEDs, to the proportion of cases with DOACs and control AEDs, such as levetiracetam.

Results: Compared with control AEDs, first-generation AEDs were associated with increased odds of reporting outcomes (reporting odds ratio {ROR}: 1.79, 95% confidence interval {CI}: 1.52-2.10, p<0.0001). Dabigatran, rivaroxaban, and apixaban were also similarly associated with increased reporting of outcome (ROR: 1.74, 95% CI: 1.26-2.39, ROR: 1.58, 95% CI: 1.24-2.02, and ROR: 2.07, 95% CI: 1.48-2.90). Edoxaban and JADER scores were not associated with patient outcomes.

Conclusion: We observed an increase in the odds of reporting anticoagulation treatment failure among patients treated with DOACs and concomitant first-generation AEDs compared to those treated with control AEDs in the FAERS database. Care should be taken when administering dabigatran, apixaban, and rivaroxaban.

## Introduction

Anticoagulants are essential for treating complications of atrial fibrillation (AF). Direct oral anticoagulants (DOACs) are recommended over warfarin in DOAC-eligible patients with AF in the 2019 guidelines for the management of patients with AF and venous thromboembolism (VTE) [1,2]. Recent advances in stroke treatment have dramatically reduced mortality rates associated with stroke. With the increased prevalence of post-stroke survivors, the number of patients with post-stroke seizures (PSS) is expected to increase [3]. PSS is a significant complication of stroke, with a reported cumulative incidence of 9.0% [4]. Additionally, mortality among stroke patients with AF is increasing as the population ages [5]. Therefore, many patients require long-term antiepileptic drug (AED) treatment. In the Medicare population, one study reported that initial AEDs, such as levetiracetam, were commonly prescribed for outpatients with PSS [6]. Therefore, it is common for some patients to undergo concomitant treatment with DOAC-AEDs, which could lead to pharmacological interactions with serious health consequences.

The gastrointestinal absorption of all DOACs is affected by P-glycoprotein (P-gp) transport [7]. The hepatic enzyme cytochrome P450 also plays an essential role in apixaban and rivaroxaban metabolism. Dabigatran etexilate, a prodrug of dabigatran and a thrombin inhibitor, is eliminated after esterase-mediated hydrolysis. The anti-Xa anticoagulants - apixaban, rivaroxaban, and edoxaban - are, in addition to being P-gp substrates, also substrates of the hepatic cytochrome P450 (CYP450) system, particularly the CYP3A4 isoform [7]. Edoxaban is only affected by CYP3A4 via a minor pathway. Therefore, drugs that induce P-gp efflux transporters and CYP450 enzymes, such as AEDs, may decrease DOAC plasma concentrations and potentially impair efficacy. Some case reports suggest that stroke and systemic embolism can be caused by concomitant treatment with DAOCs-AEDs [8]. In particular, first-generation AEDs, such as carbamazepine, have been reported to cause drug-drug interactions (DDI) with concomitant DOACs and are defined as strong inducers [9]. In contrast, Wang et al. reported that concurrent DOACs use with some AEDs was associated with an increased risk of major bleeding due to drug-drug interactions [10]. However, there is no evidence that the concurrent use of inducers in patients taking DOACs is associated with an increased risk of stroke or thromboembolic events. Investigating the relationship between inducer AEDs, particularly the strength of inducers and the risk of thromboembolism, may help guide medication selection. Therefore, this study aimed to evaluate the association between concomitant use of DOACs and inducer AEDs with thrombotic event risk using the FDA Adverse Event Reporting System (FAERS) and Japanese Adverse Drug Event Report (JADER) database. This article was previously presented as a meeting abstract at the 144th Annual Meeting of the Pharmaceutical Society of Japan on March 30, 2024.

## Materials and methods

FAERS and JADER analysis

Data Source

We analyzed the adverse event reports submitted to FAERS from January 2010 to June 2023 and JADER from January 2004 to April 2023. FAERS is a publicly accessible database that aggregates and summarizes adverse drug reaction reports from across the globe. Detailed data from FAERS were downloaded from the FDA website [11]. We primarily used the reaction (REAC) and drug (DRUG) files, which contain information about the adverse reaction and the associated drug.

The JADER database is also publicly accessible and contains information on cases reported by pharmaceutical companies and medical institutions since 2004. Data from JADER were downloaded from the pharmaceutical and medical devices agency website [12]. The JADER database comprises the following four datasets: patient statistics (DEMO), drug information (DRUG), adverse events (REAC), and medical history (HIST). The corresponding case data from the DRUG, REAC, and DEMO tables are combined using identification numbers for each adverse event. We outsourced the process to INTAGE Healthcare Inc. (Tokyo, Japan) as follows: they were combined into four datasets and extracted DOACs and AEDs in DRUG in the JADER database. The institutional review board approval is not required for this study, as JADER and FAERS data are publicly available.

Study Design

**Figure 1:**
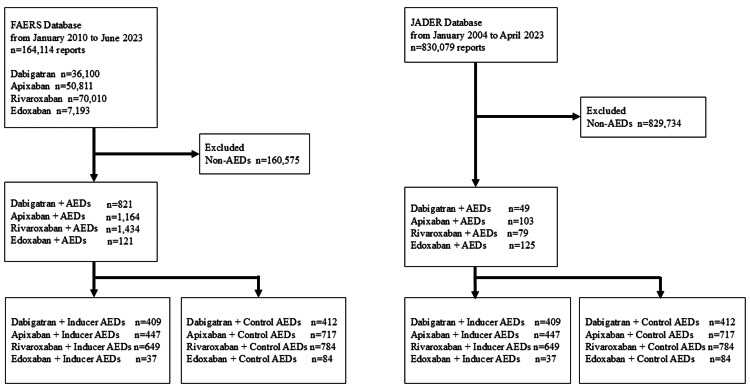
Flowchart of this study. We included 164,114 reports in the FAERS database and 356 reports in the JADER. FAERS: FDA Adverse Event Reporting System; JADER: Japanese Adverse Drug Event Report database; AED: antiepileptic drug

This retrospective, observational cohort study analyzed patients enrolled in the FAERS and JADER databases. Studies utilizing the FAERS and JADER databases have been proposed to investigate potential associations between drugs and adverse drug reactions, focusing on signal detection [13]. A flowchart of our study is shown in Figure 1.

We included 164,114 reports from the FAERS database and 356 reports from the JADER database. Reports that did not include DOACs under the “suspect product active ingredients” or “concomitant product names” in the FAERS database were excluded. Since multiple adverse drug reactions can be associated with a single case, duplicate entries were removed using the unique identifiers primary ID and case ID. This study did not adjust for potential confounders such as age, comorbidities, or concurrent medications. Therefore, cases with missing gender or age values were not excluded. Adverse events in the analysis dataset were classified according to the standards of the Medical Dictionary for Regulatory Activities (MedDRA) version 26.0. We assessed ischemic stroke or thromboembolic events associated with DOACs alone, DOACs in combination with inducer AEDs, and DOACs with control AEDs. We compared the proportion of case-reporting ischemic stroke or thromboembolic events with inducer AEDs with those with control AEDs. In addition, we reported the proportion of cases with ischemic stroke or thromboembolic events with dabigatran, rivaroxaban, apixaban, and edoxaban.

Materials

Cases about various manifestations of ischemic stroke or thromboembolic events were identified by following keywords: cerebrovascular accident, cerebrovascular disorder, cerebral infarction, stroke, transient ischemic attack, venous thrombosis, pulmonary embolism, pulmonary thrombosis, anticoagulation drug level decrease, or anticoagulation drug level below. Therefore, cases whose reports contained “embolic and thrombotic events, venous,” and “ischemic central nervous system vascular conditions” were included in our analysis. We also investigated the years of reporting, age, and sex of patients in each database. Cases involving DOACs were identified by the generic and brand names of the four approved direct oral anticoagulants as follows: dabigatran, rivaroxaban, apixaban, and edoxaban. Cases involving AEDs were identified by antiepileptic medication using the “suspect product active ingredients” and “concomitant product names” in the FAERS.

Inducer AEDs include phenytoin, phenobarbital, carbamazepine, and topiramate. In addition, inducer AEDs were classified as weak inducer AEDs (topiramate) and strong inducer AEDs (phenytoin, phenobarbital, and carbamazepine) based on observed trends. The control AEDs were lamotrigine, levetiracetam, and lacosamide. Signal detection of an interaction between DOACs and levetiracetam in combination has been shown [14], but it was included as a control because it was suggested that it may not be an important P-gp inducer in humans [15,16]. They were identified based on their generic and brand names.

Statistical analysis

Descriptive statistical analysis was performed, and categorical variables such as age and sex were represented as frequencies and percentages. The statistical analysis conducted in this study was based on data mining techniques for disproportionality analysis in pharmacovigilance [13]. A 2×2 contingency table was created to identify drug combinations that resulted in disproportionate adverse events. The reported odds ratios (RORs) were calculated as (a×d)/(b×c) and expressed as point estimates with 95% confidence intervals (CIs). A signal was identified if the lower bound of the 95% CI for the ROR exceeded 1.0 [17]. Statistical significance was determined using the chi-square test. These techniques provide quantitative measures for detecting the association between a particular drug and a particular adverse drug event. The ROR compares the odds of ischemic stroke or thromboembolic events in adverse event reports involving DOACs alone versus those involving DOACs with concomitant AEDs. In addition, the ROR compares the odds of ischemic stroke or thromboembolic events associated with DOACs and inducer AEDs to the odds associated with DOACs and control AEDs. Trends among the three groups (strong inducers, weak inducers, and the control group) were analyzed using the Cochran-Armitage trend test. All analyses were performed using the SAS University Edition software (Cary, NC: SAS Institute).

## Results

Characteristic reporting in the FAERS and JADER

The overall number of reports was the highest for rivaroxaban (1,434 reports) and edoxaban (125 reports) in the FAERS and JDAER databases, respectively (Table 1). The reporting year in the FAERS database showed a sharp increase in dabigatran use from 2011, with apixaban being the most recent. Dabigatran, apixaban, and rivaroxaban showed similar trends, whereas edoxaban was the most recently reported drug in the JADER. FAERS had a higher percentage of patients with unknown age and sex than JADER. However, the percentage of reports by age for each drug showed a similar trend, with dabigatran, apixaban, and rivaroxaban in the 70-80 years age range and edoxaban in the 80-90 years age range. The reported rate of stroke or VTE was higher among patients aged 60-90 years. Except for edoxaban, stroke or VTE was more frequently reported in men.

**Table 1:** Reporting baseline characteristics in the FAERS and JADER. FAERS: FDA Adverse Event Reporting System; JADER: Japanese Adverse Drug Event Report; VTE: venous thromboembolism

FAERS									JADER								
Reporting year	Dabigatran (n=821)		Apixaban (n=1,164)		Rivaroxaban (n=1,434)		Edoxaban (n=121)		Reporting year	Dabigatran (n=49)		Apixaban (n=103)		Rivaroxaban (n=79)		Edoxaban (n=125)	
	All reports, n (%)	Stroke or VTE reports, n (%)	All reports, n (%)	Stroke or VTE reports, n (%)	All reports, n (%)	Stroke or VTE reports, n (%)	All reports, n (%)	Stroke or VTE reports, n (%)		All reports, n (%)	Stroke or VTE reports, n (%)	All reports, n (%)	Stroke or VTE reports, n (%)	All reports, n (%)	Stroke or VTE reports, n (%)	All reports, n (%)	Stroke or VTE reports, n (%)
2020-2023	127 (15.5)	-	614 (52.7)	-	310 (21.6)	-	78 (64.5)	-	2020-2023	7 (14.3)	-	33 (32.0)	-	23 (29.1)	-	65 (52.0)	-
2017-2019	249 (30.3)	-	395 (33.9)	-	427 (29.8)	-	38 (31.4)	-	2017-2019	21 (42.9)	-	41 (39.8)	-	23 (29.1)	-	39 (31.2)	-
2014-2016	189 (23.0)	-	143 (12.3)	-	585 (40.8)	-	5 (4.1)	-	2014-2016	10 (20.4)	-	28 (27.2)	-	26 (32.9)	-	21 (16.8)	-
2011-2013	251 (30.6)	-	7 (0.6)	-	110 (7.7)	-	0 (0.0)	-	2011-2013	11 (22.4)	-	1 (1.0)	-	7 (8.9)	-	0 (0.0)	-
<2010	4 (0.5)	-	5 (0.5)	-	0 (0.0)	-	0 (0.0)	-	<2010	0 (0.0)	-	0 (0.0)	-	0 (0.0)	-	0 (0.0)	-
Age (years)																	
<50	49 (6.0)	11 (22.4)	109 (9.4)	11 (10.1)	224 (15.6)	55 (24.6)	8 (6.6)	2 (25.0)	<50	0 (0.0)	0 (0.0)	2 (1.9)	0 (0.0)	6 (7.6)	1 (16.7)	11 (8.8)	1 (9.1)
50-60	60 (7.3)	11 (18.3)	68 (5.8)	10 (14.7)	185 (12.9)	36 (19.5)	3 (2.5)	1 (33.3)	50-60	2 (4.1)	0 (0.0)	4 (3.9)	0 (0.0)	5 (6.3)	0 (0.0)	13 (10.4)	2 (15.4)
60-70	155 (18.9)	41 (26.5)	193 (16.6)	17 (8.8)	293 (20.4)	72 (24.6)	6 (5.0)	2 (33.3)	60-70	14 (28.6)	2 (14.3)	15 (14.6)	0 (0.0)	9 (11.4)	1 (11.1)	18 (14.4)	2 (11.1)
70-80	246 (30.0)	67 (27.2)	262 (22.5)	51 (19.5)	304 (21.2)	60 (19.7)	15 (12.4)	3 (20.0)	70-80	16 (32.7)	0 (0.0)	45 (43.7)	2 (4.4)	30 (38.0)	4 (13.3)	34 (27.2)	3 (8.8)
80-90	177 (21.6)	47 (26.6)	250 (21.5)	44 (17.6)	192 (13.4)	56 (29.2)	23 (19.0)	4 (17.4)	80-90	15 (30.6)	1 (6.7)	32 (31.1)	4 (12.5)	25 (31.6)	1 (4.0)	39 (31.2)	3 (50.0)
90≤	15 (1.8)	3 (20.0)	46 (4.0)	4 (8.7)	35 (2.4)	13 (37.1)	10 (8.3)	1 (10.0)	90≤	1 (2.0)	0 (0.0)	4 (3.9)	0 (0.0)	4 (5.1)	0 (0.0)	6 (4.8)	
Unknown	113 (13.8)	-	232 (19.9)	-	193 (13.5)	-	56 (46.3)	-	Unknown	1 (2.0)	-	1 (1.0)	-	0 (0.0)	-	4 (3.2)	-
Sex																	
Male	432 (52.6)	116 (26.9)	516 (44.3)	85 (16.5)	638 (44.5)	175 (27.4)	48 (39.7)	7 (14.6)	Male	35 (71.4)	3 (8.6)	55 (53.4)	4 (7.3)	52 (65.8)	5 (9.6)	65 (52.0)	10 (15.4)
Female	364 (44.3)	85 (23.4)	604 (51.9)	63 (10.4)	717 (50.0)	133 (18.5)	33 (27.3)	13 (36.4)	Female	13 (26.5)	0 (0.0)	48 (46.6)	2 (4.2)	27 (34.2)	2 (7.4)	60 (48.0)	5 (8.3)
Unknown	25 (3.0)	-	44 (3.8)	-	78 (5.4)	-	40 (33.1)	-	Unknown	1 (2.0)	-	0 (0.0)	-	0 (0.0)	-	0 (0.0)	-

FAERS Analysis

During the review period, 164,114 reports included DOACs. The proportion of reports of stroke or VTE was higher among patients treated with AEDs (23.1%) than among patients treated with dabigatran alone (11.9%) (reporting odds ratio {ROR}: 2.22; 95% confidence interval {CI}: 1.89-2.62) (Table 2). Similar findings were observed with apixaban and rivaroxaban.

**Table 2:** Stroke or VTE event reports and all other adverse event reports with each DOACs only and DOACs concomitant AEDs in FAERS. AEDs were defined as carbamazepine, phenytoin, phenobarbital, topiramate, lamotrigine, levetiracetam and lacosamide. DOAC: direct oral anticoagulants; AED: antiepileptic drug; VTE: venous thromboembolism; ROR: reporting odds ratio; CI: confidence interval

Variables	FAERS				
	Stroke or VTE reports, n (%)	Total, n (%)	ROR	95% CI	p-Value
Dabigatran	-	-	2.22	1.89-2.62	<0.0001
Dabigatran	4,206 (11.9)	35,279 (97.7)	-	-	-
Dabigatran+AEDs	190 (23.1)	821 (2.3)	-	-	-
Apixaban	-	-	2.33	1.96-2.77	<0.0001
Apixaban	3,053 (6.2)	49,647 (97.7)	-	-	-
Apixaban+AEDs	154 (13.2)	1,164 (2.3)	-	-	-
Rivaroxaban	-	-	2.29	2.01-2.60	<0.0001
Rivaroxaban	7,382 (10.8)	68,577 (98.0)	-	-	-
Rivaroxaban+AEDs	310 (21.6)	1,433(2.0)	-	-	-
Edoxaban	-	-	1.31	0.84-2.02	0.2531
Edoxaban	1,225 (17.3)	7,072 (98.3)	-	-	-
Edoxaban+AEDs	26 (21.5)	121 (1.7)	-	-	-

During the review period, 3,539 reports included concomitant DOACs, inducer AEDs, and control AEDs. Almost all reports used dabigatran (9.1%), rivaroxaban (40.5%), and apixaban (32.9%), and edoxaban was used in only 121 (3.4%) cases (Table 3).

**Table 3:** Baseline characteristics included in FAERS and JADER. Inducer AEDs were defined as carbamazepine, phenytoin, phenobarbital, and topiramate. All AEDs were defined as carbamazepine, phenytoin, phenobarbital, topiramate, lamotrigine, and levetiracetam. Inhibitor drugs were defined as strong CYP3A4 inhibitors, moderate CYP3A4 inhibitors, inhibitors of P-gp, and antiplatelet drugs. FAERS: FDA Adverse Event Reporting System; JADER: Japanese Adverse Drug Event Report database; AED: antiepileptic drug

FAERS	Inducer AEDs (n=1,542)	All AEDs (n=3,539)
Dabigatran, n (%)	409 (49.8)	821 (9.1)
Apixaban, n (%)	447 (38.4)	1,164 (32.9)
Rivaroxaban, n (%)	649 (45.3)	1,433 (40.5)
Edoxaban, n (%)	37 (29.8)	121 (3.4)
JADER	Inducer AEDs (n=147)	All AEDs (n=345)
Dabigatran, n (%)	22 (15.0)	49 (14.2)
Apixaban, n (%)	65 (44.2)	103 (29.9)
Rivaroxaban, n (%)	38 (25.9)	79 (22.9)
Edoxaban, n (%)	43 (55.8)	125 (36.2)

The outcomes of concomitant use of DOACs and all AEDs were reported in 739 reports. The proportion of reports involving outcomes was higher among patients treated with inducer AEDs (26.3%) than among patients treated with control AEDs (16.7%) (reporting odds ratio {ROR}: 1.79, 95% confidence interval {CI}: 1.52-2.10) (Table 4).

**Table 4:** Stroke or VTE event reports and all other adverse event reports with each DOACs in FAERS and JADER. *Inducer AEDs were defined as carbamazepine, phenytoin, phenobarbital, and topiramate. **Control AEDs were defined agents as lamotrigine, levetiracetam, and lacosamide. DOAC: direct oral anticoagulants; AED: antiepileptic drug; VTE: venous thromboembolism; ROR: reporting odds ratio; CI: confidence interval

Variables	FAERS					JADER				
	Stroke or VTE reports, n (%)	Total, n (%)	ROR	95% CI	p-Value	Stroke or VTE reports, n (%)	Total n (%)	ROR	95% CI	p-Value
DOACs	-	-	1.79	1.52-2.10	<0.0001	-	-	0.997	0.93-1.07	0.9367
Inducer AEDs^*^	406 (26.3)	1,542 (43.6)	-	-	-	13 (3.8)	147 (42.6)	-	-	-
Control AEDs^**^	333 (16.7)	1,997 (56.4)	-	-	-	18 (5.2)	198 (57.4)	-	-	-
Dabigatran	-	-	1.74	1.26-2.39	0.0006	-	-	NA	NA	NA
Inducer AEDs^*^	126 (30.8)	409 (49.8)	-	-	-	3 (11.1)	27 (55.1)	-	-	-
Control AEDs^**^	84 (20.4)	412 (50.2)	-	-	-	0 (0.0)	22 (44.9)	-	-	-
Apixaban	-	-	2.07	1.48-2.90	<0.0001	-	-	0.96	0.86-1.07	0.4679
Inducer AEDs^*^	86 (19.2)	447 (38.4)	-	-	-	2 (5.3)	38 (36.9)	-	-	-
Control AEDs^**^	74 (10.3)	717 (61.6)	-	-	-	6 (9.2)	65 (63.1)	-	-	-
Rivaroxaban	-	-	1.58	1.24-2.02	0.0002	-	-	1.02	0.89-1.17	0.7711
Inducer AEDs^*^	181 (27.9)	649 (45.3)	-	-	-	4 (9.8)	41 (51.9)	-	-	-
Control AEDs^**^	154 (19.6)	784 (54.7)	-	-	-	3 (7.9)	38 (48.1)	-	-	-
Edoxaban	-	-	1.63	0.70-3.75	0.2531	-	-	0.95	0.84-1.09	0.5015
Inducer AEDs^*^	13 (35.1)	37 (30.6)	-	-	-	4 (9.3)	43 (34.4)	-	-	-
Control AEDs^**^	21 (25.0)	84 (69.4)	-	-	-	11 (13.4)	82 (65.6)	-	-	-

In addition, dabigatran, apixaban, and rivaroxaban showed a trend toward higher rates of stroke or VTE with increasing inducer strength among the three groups (all p-values for trends <0.001) (Table 5).

**Table 5:** Stroke or VTE event reports and all other adverse event reports with each DOAC and inducer strength AED in FAERS. *Strong inducer AEDs were defined as carbamazepine, phenytoin, and phenobarbital. Weak Inducer AEDs were defined as topiramate. **Control AEDs were defined agents as lamotrigine, levetiracetam, and lacosamide. DOAC: direct oral anticoagulants; AED: antiepileptic drug; VTE: venous thromboembolism; ROR: reporting odds ratio; CI: confidence interval; FAERS: FDA Adverse Event Reporting System

FAERS		
Variables	Stroke or VTE reports, n (%)	Total, n (%)
Dabigatran		
Strong inducer AEDs^*^	111 (33.7)	329 (40.1)
Weak inducer AED	15 (18.8)	80 (9.7)
Control AEDs^**^	84 (20.4)	412 (50.2)
Apixaban		
Strong inducer AEDs^*^	75 (21.8)	343 (29.5)
Weak inducer AED	11 (10.6)	104 (8.9)
Control AEDs^**^	74 (10.3)	717 (61.6)
Rivaroxaban		
Strong inducer AEDs^*^	145 (34.1)	425 (29.7)
Weak inducer AED	36 (16.1)	224 (15.6)
Control AEDs^**^	154 (19.6)	784 (54.7)
Edoxaban		
Strong inducer AEDs^*^	13 (35.1)	37 (30.6)
Weak inducer AED	0 (0.0)	0 (0.0)
Control AEDs^**^	21 (25.0)	84 (69.4)

JADER Analysis

During the review period, 345 studies reported on the use of DOACs and AEDs. Of these, 147 reports included concomitant use of DOACs and first-generation AEDs, and 198 included concomitant DOACs and control AEDs. All reports included dabigatran (14.2%), rivaroxaban (22.9%), apixaban (29.9%), and edoxaban (36.2%) (Table 4).

Outcomes were reported in 31 reports of concomitant use of DOACs and all AEDs (9.0%). The proportion of reports involving outcomes did not differ between first-generation AEDs (3.8%) and control AEDs (5.2%) (reporting odds ratio {ROR}: 0.997, 95% CI: 0.93-1.07) (Table 3). First-generation AEDs did not differ for each DOAC (apixaban: ROR: 0.96, 95% CI: 0.86-1.07, rivaroxaban: ROR: 1.02, 95% CI: 0.89-1.17, and edoxaban: ROR: 0.95, 95% CI: 0.84-1.09, respectively) (Table 3). Dabigatran had no data. These criteria were based on reports that included both DOACs and first-generation AEDs.

## Discussion

Characteristic reporting in the FAERS and JADER

Dabigatran was the first DOAC approved in the United States and Japan. Edoxaban was developed in Japan and was the first Xa inhibitor approved for most indications. In contrast, rivaroxaban has the most indications in the United States. Margraff and Bertram reported that adverse effects reported by patients represented an average of 9% (91% were from healthcare professionals), with the highest patient reporting rates observed in the United States (48%) [18]. This result may be reflected in the difference in the ratio of the number of cases reported by the FAERS and JADER. Stroke tends to occur more frequently in men at younger ages, but this trend reverses in women at older ages [19]. Additionally, women are reported to have a lower incidence of VTE compared to men [20]. In this study, the incidence of stroke or VTE tended to be higher in men than in women in terms of the number of reports, which may have contributed to the higher number of VTE cases observed.

FAERS and JADER analysis

This study found that patients who concomitantly used DOACs and inducer AEDs were more likely to experience failure of anticoagulation therapy compared to control AED users. In addition, the finding that edoxaban did not produce signals compared with apixaban, rivaroxaban, and dabigatran is noteworthy. Stroke and VTE events were rare outcomes due to the reporting of 1,116 stroke or systemic embolism events in 79,302 DOAC-treated patients with AF and 118,124 patient-years of follow-up (0.94/100 patient-years) [21]. However, the outcome is clinically important, as patients with both AF and VTE take DOACs for an extended period of time.

All DOACs are substrates for the efflux of P-gp transporters. Rivaroxaban and apixaban also depend on cytochrome P450 metabolism. Although CYP3A4 is related to the metabolic processes of edoxaban, it is minimal (10%), and dabigatran does not undergo CYP metabolism [22]. In typical use, edoxaban has drug-drug interactions, which might influence fewer metabolic pathways than apixaban and rivaroxaban in CYP3A4. Di Gennaro et al. reported a significant interaction between carbamazepine and apixaban in patients with AF who had a transient ischemic attack (TIA), but edoxaban administered to patients after TIA occurrence did not show a significant interaction with carbamazepine [23]. We believe that this result may be due to the differences in their pharmacokinetic profiles. Edoxaban was approved for the prevention of VTE after major orthopedic surgery in Japan, the first approval worldwide in 2011. Therefore, the number of edoxaban event reports is greater than that of other DOACs in the JADER database than in the FAERS database. In fact, all studies in the systematic review of the edoxaban group were very small [8].

Steffel et al. reported that for patients with AF, concomitant use of strong inducers such as phenytoin was not approved [24]. In our study, the number of reported stroke cases tended to be higher for the more strongly inducing AEDs, which is consistent with these reports. However, the steps that should be taken to minimize the risk of treatment failure in patients with AF using this drug combination remain unclear. DOACs were approved at a fixed dose that did not require time-division multiplexing while achieving effective and safe anticoagulation. However, these promises have only been partially fulfilled. According to these guidelines, drug-level measurements can guide therapy in patients where significant drug interactions cannot be avoided; drug-level measurements can guide therapy. However, the safe and effective drug level ranges of DOACs have not been clearly defined or widely accepted. Conversely, Acton et al. reported that compared with the use of non-enzyme-inducing (EI) antiseizure medications (ASMs), the use of EI ASMs with DOACs was not associated with a difference in the risk of thromboembolic events [25]. Thus, the significance of these DDIs is still largely unknown, with only occasional case reports available [8]. In addition, the available data point to a possible increased risk of thrombotic events between DOACs and AEDs; however, they are insufficient to draw definitive conclusions for each DOAC [8].

DOACs have been reported to be effective and safe in several large clinical trials for AF and VTE [26]. The incidence of stroke or VTE in patients with atrial fibrillation is less than approximately 2% per year [26]. However, in patients not receiving anticoagulants, the risk of such events exceeds 5% [27]. PSS patients require AEDs to control seizures in addition to taking DOACs. Therefore, it is important to understand the characteristics of these drugs before selecting an AED. Although current guidelines lack robust recommendations for treating PSS and epilepsy, a prospective cross-sectional single-center study reported that lacosamide, when used in combination with an NOAC in patients with post-stroke epilepsy and AF, did not increase the risk of ischemic events [28]. Additionally, a study in Australia reported a shift toward the use of newer-generation AEDs, such as levetiracetam and valproate, as well as carbamazepine, for PSS and epilepsy [29]. Therefore, we believe that our future research utilizing large-scale healthcare claims data may help to further clarify these issues.

Our study had several limitations. It was based on the FAERS and JADER databases and is susceptible to confounding variables such as patient comorbidities, concurrent medications, and lifestyle factors that are not adequately controlled for, as well as bias. In particular, reliance on spontaneous adverse event reports introduces reporting bias, as underreporting and selective reporting may affect the accuracy of the findings. Additionally, we could not fully address issues such as case duplications, differences in coding practices, lack of adjusted analyses, and lack of a true denominator. While this study establishes an association between DOAC-AED interactions and thrombotic events, it does not confirm a direct causal relationship. Furthermore, edoxaban, which had relatively few reports, may require further investigation. Although the FAERS and JADER databases contain extensive data on real-world adverse outcomes, they provide limited information on patient characteristics such as age, weight, doses, adherence, liver function, renal function, or potential drug interactions. However, these studies did not include laboratory measurements. Therefore, drug levels among patients with and without drug interactions cannot be used to support a causal mechanism for the observed associations. It is also difficult to control for factors that may confound the relationship between medication and outcome occurrence because factors such as the dose of the drug used and renal function cannot be determined.

## Conclusions

This study suggests that the concomitant use of dabigatran, rivaroxaban, apixaban, P-gp, and enzyme-inducing AEDs may reduce the treatment efficacy and increase the risk of serious thrombotic and stroke events. On the other hand, although no signal was detected for edoxaban, this may be due to its different pharmacokinetic profile. However, the available data were insufficient to exclude potential DDI. The current evidence is limited and statistically underpowered to draw definitive conclusions regarding clinical significance. Therefore, well-designed clinical and epidemiological studies are warranted.

## References

[1] January CT, Wann LS, Calkins H (2019). 2019 AHA/ACC/HRS focused update of the 2014 AHA/ACC/HRS guideline for the management of patients with atrial fibrillation: a report of the American College of Cardiology/American Heart Association Task Force on Clinical Practice Guidelines and the Heart Rhythm Society in collaboration with the Society of Thoracic Surgeons. Circulation.

[2] Ortel TL, Neumann I, Ageno W (2020). American Society of Hematology 2020 guidelines for management of venous thromboembolism: treatment of deep vein thrombosis and pulmonary embolism. Blood Adv.

[3] Krueger H, Koot J, Hall RE, O'Callaghan C, Bayley M, Corbett D (2015). Prevalence of individuals experiencing the effects of stroke in Canada: trends and projections. Stroke.

[4] Isah FM, Wang S, Djatche WH, Song M, Li CX (2025). Incidence and influencing factors of post-stroke seizure after endovascular treatment of ischemic stroke - a systematic review and meta-analysis. Clin Neurol Neurosurg.

[5] Khan SA, Assad AA, Ashraf H (2025). National trends in mortality due to ischemic stroke among older adults with atrial fibrillation in the USA, 1999-2020. Clin Cardiol.

[6] Donahue MA, Brooks JD, Hsu J (2025). Differences in patterns of outpatient epilepsy-specific medication initiation after acute ischemic stroke in the Medicare population. Epilepsia.

[7] Ferri N, Colombo E, Tenconi M, Baldessin L, Corsini A (2022). Drug-drug interactions of direct oral anticoagulants (DOACs): from pharmacological to clinical practice. Pharmaceutics.

[8] Candeloro M, Carlin S, Shapiro MJ, Douketis JD (2023). Drug-drug interactions between direct oral anticoagulants and anticonvulsants and clinical outcomes: a systematic review. Res Pract Thromb Haemost.

[9] Chadha A, Guirguis M, Bungard TJ (2022). DOAC drug interactions management resource. Can Pharm J (Ott).

[10] Wang CL, Wu VC, Chang KH (2020). Assessing major bleeding risk in atrial fibrillation patients concurrently taking non-vitamin K antagonist oral anticoagulants and antiepileptic drugs. Eur Heart J Cardiovasc Pharmacother.

[11] (2025). FDA Adverse Event Reporting System (FAERS) Public Dashboard. https://www.fda.gov/drugs/fdas-adverse-event-reporting-system-faers/fda-adverse-event-reporting-system-faers-public-dashboard.

[12] (2025). Drug adverse reaction database terms of use. https://www.pmda.go.jp/safety/info-services/drugs/adr-info/suspected-adr/0003.html.

[13] Poluzzi E, Raschi E, Piccinni C, De Ponti F (2012). Data mining techniques in pharmacovigilance: analysis of the publicly accessible FDA Adverse Event Reporting System (AERS). Data Mining Applications in Engineering and Medicine.

[14] Kaoud MA, Nissan R, Segev A, Sabbag A, Orion D, Maor E (2023). Levetiracetam interaction with direct oral anticoagulants: a pharmacovigilance study. CNS Drugs.

[15] Mavri A, Ilc S (2023). The efficacy of direct oral anticoagulants in patients on concomitant treatment with levetiracetam. Sci Rep.

[16] Rota E, Immovilli P, Pappalardo I (2024). Direct oral anticoagulants and concomitant anti-seizure medications: a retrospective, case-control study in a real-world setting. Clin Ther.

[17] Cutroneo PM, Sartori D, Tuccori M (2024). Conducting and interpreting disproportionality analyses derived from spontaneous reporting systems. Front Drug Saf Regul.

[18] Margraff F, Bertram D (2014). Adverse drug reaction reporting by patients: an overview of fifty countries. Drug Saf.

[19] Cherian L (2023). Women and ischemic stroke: disparities and outcomes. Neurol Clin.

[20] Afifi AM, Leverich M, Tadrousse K, Ren G, Nazzal M (2024). Racial, biological sex, and geographic disparities of venous thromboembolism in the United States, 2016 to 2019. J Vasc Surg Venous Lymphat Disord.

[21] Gronich N, Stein N, Muszkat M (2021). Association between use of pharmacokinetic-interacting drugs and effectiveness and safety of direct acting oral anticoagulants: nested case-control study. Clin Pharmacol Ther.

[22] Parasrampuria DA, Truitt KE (2016). Pharmacokinetics and pharmacodynamics of edoxaban, a non-vitamin K antagonist oral anticoagulant that inhibits clotting factor Xa. Clin Pharmacokinet.

[23] Di Gennaro L, Lancellotti S, De Cristofaro R, De Candia E (2019). Carbamazepine interaction with direct oral anticoagulants: help from the laboratory for the personalized management of oral anticoagulant therapy. J Thromb Thrombolysis.

[24] Steffel J, Verhamme P, Potpara TS (2018). The 2018 European Heart Rhythm Association Practical Guide on the use of non-vitamin K antagonist oral anticoagulants in patients with atrial fibrillation. Eur Heart J.

[25] Acton EK, Hennessy S, Gelfand MA (2024). Direct-acting oral anticoagulants and antiseizure medications for atrial fibrillation and epilepsy and risk of thromboembolic events. JAMA Neurol.

[26] Sharma M, Cornelius VR, Patel JP, Davies JG, Molokhia M (2015). Efficacy and harms of direct oral anticoagulants in the elderly for stroke prevention in atrial fibrillation and secondary prevention of venous thromboembolism: systematic review and meta-analysis. Circulation.

[27] Connolly SJ, Laupacis A, Gent M, Roberts RS, Cairns JA, Joyner C (1991). Canadian Atrial Fibrillation Anticoaguiation (CAFA) study. J Am Coll Cardiol.

[28] Mangiardi M, Pezzella FR, Cruciani A, Alessiani M, Anticoli S (2024). Long-term safety and efficacy of lacosamide combined with NOACs in post-stroke epilepsy and atrial fibrillation: a prospective longitudinal study. J Pers Med.

[29] Kim SJ, Wood S, Marquina C, Foster E, Bell JS, Ilomäki J (2023). Shift from older- to newer-generation antiseizure medications in people with acute ischemic stroke in Australia: a population-based study. Epilepsia Open.

